# Spectroscopic, Thermal and Biological Studies on Some Trivalent Ruthenium and Rhodium NS Chelating Thiosemicarbazone Complexes

**DOI:** 10.1155/2007/68374

**Published:** 2007-03-06

**Authors:** Vinod K. Sharma, Shipra Srivastava, Ankita Srivastava

**Affiliations:** Department of Chemistry, Faculty of Science, University of Lucknow, Lucknow 226007, India

## Abstract

The synthetic, spectroscopic, and biological studies of sixteen ring-substituted 4-phenylthiosemicarbazones and 4-nitrophenyl-thiosemicarbazones of anisaldehyde, 4-chlorobenzaldehyde, 4-fluorobenzaldehyde, and vanillin with ruthenium(III) and rhodium(III) chlorides are reported here. Their structures were determined on the basis of the elemental analyses, spectroscopic data (IR, electronic, ^1^H and ^13^C NMR) along with magnetic susceptibility measurements, molar conductivity and thermogravimetric analyses. Electrical conductance measurement revealed a 1 : 3 electrolytic nature of the complexes. The resulting colored products are monomeric in nature. On the basis of the above studies, three ligands were suggested to be coordinated to each metal atom by thione sulphur and azomethine nitrogen to form low-spin octahedral complexes with ruthenium(III) while forming diamagnetic complexes with rhodium(III). Both ligands and their complexes have been screened for their bactericidal activities and the results indicate that they exhibit a significant activity.

## 1. INTRODUCTION

**Figure 1:**
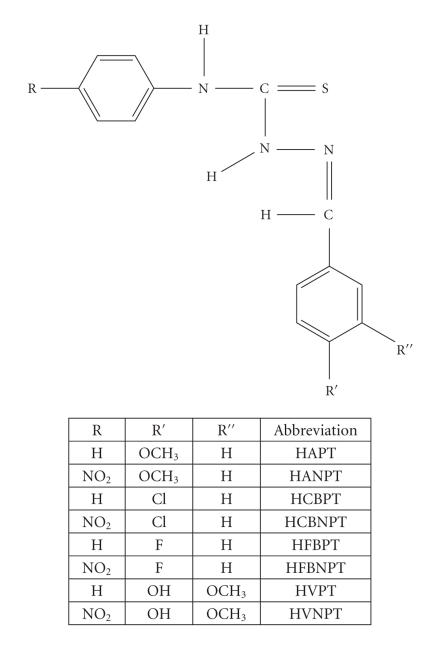
Structure of the thiosemicarbazone ligands.

The synthesis and structural investigations of thiosemicarbazones and their metal complexes are of considerable centre of attention because of their potentially beneficial pharmacological properties and a wide variation in their modes of bonding and stereochemistry [1–3]. Coordination chemistry of mixed hard-soft NS donor ligands is a field of current interest. The most important factor in this objective is probably the design of ligands with an appropriate structural backbone. Thiosemicarbazones that are most widely studied are sulphur and nitrogen consisting ligands [4, 5]. Besides, thiosemicarbazones have emerged as an important sulphur containing ligands in the last two decades [6–9]. The real impetus towards coordination chemistry is the wide range of biological properties depending on parent aldehyde or ketone including antitumour [10, 11], antibacterial, and antifungal [12, 13] properties as well as their physicochemical effects [14, 15]. In addition of this, they have been screened for their medicinal properties because they possess some cytotoxic effect. They also stabilize uncommon oxidation states, generate a different coordination number in transition metal complexes in order to participate in various redox reactions [16, 17]. It is well known that several metal ions enhance and modify the biological activities of thiosemicarbazones, the new metals to such a list are ruthenium [18] and rhodium [19]. Much attention has been drawn towards the chemistry of ruthenium [20, 21] and rhodium [22] in different coordination spheres. Due to different oxidation states of ruthenium and rhodium their reactivity depends upon stability of oxidation states. In view of this ruthenium(III), thiosemicarbazones with nitrogen and sulphur as donor atoms have been found to be very efficient catalysts in the oxidation of alcohols and alkenes [23]. With the growing interest of thiosemicarbazones of ruthenium and rhodium metal ions, here we report the synthesis, characterization, and biological activities of the ruthenium(III) and rhodium(III) thiosemicarbazones obtained by condensation of the ring-substituted 4-phenylthiosemicarbazide and 4-nitrophenylthiosemicarbazide with anisaldehyde, 4-chlorobenzaldehyde, 4-fluorobenzaldehyde, and vanillin. Biological activities of the complexes and ligands have also been carried out against bacteria *Bacillus subtilis* and *Pseudomonos aeruginosa* in vitro. The ligands used in the study are of the type depicted in Figure 1.

## 2. EXPERIMENTAL

### 2.1. Materials

RuCl_3_ · 3H_2_O and RhCl_3_ · 3H_2_O and other chemicals were purchased from Merck and Loba chemie, Bombay, India, and were used without further purification.

The antibacterial activity of the ligands and their complexes were tested by using paper disc diffusion method [24] against *Bacillus subtilis* and *Pseudomonas aeruginosa*. Nutrient agar medium was prepared by using peptone, beef extract, NaCl, agar-agar, distilled water, and 5 mm diameter paper discs (whatman No.1) were used. The test organisms were dissolved in ethanol to a concentrations of 1000 and 500 ppm and soaked in filter paper discs of 5 mm diameter and 1 mm in thickness. These paper discs were kept in a petri dishes (well sterilized) previously seeded with test organisms. The plates were incubated for 24–30 hours at 28 ± 2°C. The zone of inhibition was calculated in mm carefully. Streptomycin was used as standard. The composition of test media is the factor, which often exerts the greatest effect upon the drug activity. This is particularly true in the case of thiosemicarbazones.

### 2.2. Synthesis of the ligands

4-phenylthiosemicarbazide and 4-nitrophenylthiosemicar-bazide were prepared from the appropriate amines by using a standard method [25]. The thiosemicarbazone ligands (Figure 1) were prepared by equimolar quantities of 0.02 mol of each anisaldehyde (2.72 g), 4-chlorobenzaldehyde (2.80 g), 4-fluorobenzaldehyde (2.48 g) and vanillin (3.04 g) in 10 mL ethanol with an ethanolic solution (25 mL) of 4-phenyl thiosemicarbazide (3.34 g, 0.02 mol)/4-nitrophenyl-thiosemicarbazide (4.24 g, 0.02 mol). The reaction mixtures were then refluxed on a water bath for 1 hour. Few drops of acetic acid were added during reflux. As precipitate appeared, the reaction mixture was allowed to reflux more along with stirring for 2 hours. The residue formed was separated out, filtered off, washed several times with water, recrystallized from ethanol, and finally dried in vacuo over fused calcium chloride. The proposed chemical structures of the thiosemicarbazone ligands are known to be in good agreement with the ratios concluded from analytical data (see Table 1).

### 2.3. Synthesis of the complexes

[M(L)_3_]Cl_3_ *(*M = Ru(III), Rh(III); L = HAPT, HCBPT, HFBPT *and* HVPT*)*

Hydrated RuCl_3_ (0.261 g, 0.001 mol) and RhCl_3_ (0.263 g, 0.001 mol) in ethanol (10 mL) were heated, then metal trichloride solution was suspended in 0.003 mol of each ligand viz. HAPT (2.56 g), HCBPT (2.60 g), HFBPT (2.45 g), and HVPT (2.70 g) in ethanol (20 mL). The reaction mixtures were refluxed for 9–12 hours. The precipitates formed were cooled, filtered off, and washed with hot water, hot ethanol, and finally with diethyl ether, and dried in vacuo over fused calcium chloride. The yields were 60–70%.

[M(L)_3_]Cl_3_ *(*M = Ru(III), Rh(III); L = HANPT, HCBNPT, HFBNPT *and* HVNPT*)*

Hydrated RuCl_3_ (0.261 g, 0.001 mol) and RhCl_3_ (0.263 g, 0.001 mol) in ethanol (10 mL) were suspended in 20 mL ethanolic solution of the ligands viz. HANPT (2.97 g), HCBNPT (3.00 g), HFBNPT (2.86 g), and HVNPT (3.11 g). The reaction mixtures were heated for few minutes, one equivalent of ethanolic solution of the NaOH was added and the reaction mixtures were refluxed for 9-10 hours. The compound, which was precipitated out, was filtered off, washed with water, cold ethanol, and diethyl ether, and dried in vacuo over fused calcium chloride. The yields were 60–76%.

### 2.4. Analyses

Microanalyses were performed at Elementar Vario III Carlo Erba 1108 in Central Drug Research Institute, Lucknow, India. IR spectra of the ligands and their complexes have been recorded in KBr pellets at Shimadzu FTIR 8201 spectrophotometer in 4000–200 cm^−1^. Electronic spectra of the complexes were recorded in CHCl_3_ with a Perkin Elmer Lambda 15 UV/Vis spectrophotometer. ^1^ H and ^13^ C NMR were obtained with a Bruker DRX 300 spectrometer in CDCl_3_ using TMS as standard. Sulphur was estimated gravimetrically as BaSO_4_. The percentage of nitrogen was estimated by Kjeldahl method. Magnetic susceptibility measurements on powder form of the complexes were recorded with a Gouy's balance by using mercuric tetrathiocyanato cobaltate(II) as a calibrant at 25°C. Molar conductance was carried out in 10^−3^ M solution of DMF. Thermogravimetric analyses were obtained at 10°C min^−1^ in the 25–750°C using a Shimadzu TGA-50 H analyzer. A standard method was used for determining metal ions and chlorides volumetrically and gravimetrically [26].

## 3. RESULTS AND DISCUSSION

**Figure 2:**
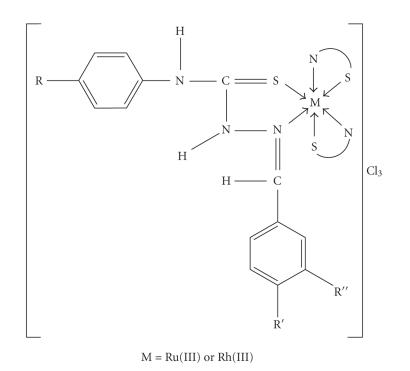
Suggested structure of the complexes.

The complexes were synthesized by reacting ligands with metal ions in 3 : 1 molar ratio in ethanolic medium. Thiosemicarbazones were expected to behave as a bidentate with sulphur and nitrogen as donor atoms or coordination sites (see Figure 2). The present thiosemicarbazone ligands exist as the thione form since it has −NH−C=S thioamide group; although, in many instances, thiol form or equilibrium mixture of both forms has been observed in thiosemicarbazones. All the ruthenium(III) complexes being d^5^ (low spin), S = 1/2 behave as paramagnetic and rhodium(III) complexes being d^6^ (low spin), S = 0 act as diamagnetic. The analytical data, magnetic susceptibility, and spectral analyses agree well with the proposed composition of formed complexes. All the complexes have shown good solubility in all the common organic solvents but were found insoluble in ether, water, acetone, and benzene. All the complexes are amorphous powder, stable at room temperature and do not show any decomposition on standing for several months. The molar conductance of the complexes in DMF lies in the range 280–315 Ω^−1^ cm^2^mol^−1^ indicating their electrolytic behavior and confirms the ionic nature of the chloride ion. Thus the complexes may be formulated as [M(L)_3_]Cl_3_ (where M = Ru(III), Rh(III); L = HAPT, HANPT, HCBPT, HCBNPT, HFBPT, HFBNPT, HVPT, and HVNPT).

The presence of chloride ions in outer sphere was tested both qualitatively and quantitatively and found very positive.

## 4. INFRARED SPECTRA

The tentative infrared absorption frequencies of the ligands and their metal complexes along with their assignments are listed in Table 2. The ligands can act either in keto or in enolic form, depending upon the conditions (e.g., pH of the medium, oxidation state of the metal ion). All physicochemical properties of the complexes support bidentate chelation of the ligands by the azomethine nitrogen and by thione sulphur. This fact was further supported by the bands including azomethine nitrogen *ν*(C=N) at 1610–1594 cm^−1^ in ligands and the lowering of this band in complexes results in chelation of the nitrogen to metal ion [27, 28]. A medium band at 1030–1020 cm^−1^ which is assigned to *ν*(N−N) in ligands is shifted to the higher frequency in the spectra of all complexes. This kind of shift on hydrazinic nitrogen described the presence of electron withdrawing substituents [29]. However, in metal complexes the band shifts to higher wave number and splits, which is probably the result of the increase in the multiplicity of the C−N bond. A strong band at 872–827 cm^−1^ in ligands is mainly due to the *ν*(C=S) stretching vibration which shifted towards lower frequency and occurred at 860–820 cm^−1^ in metal complexes indicating the coordination of thione sulphur to metal atom [30]. This also described a considerable change in bond order and a metal-sulphur bond. As the *ν*(S−H) band also remains absent, this confirms thione form of the ligand. In ligands as well as in complexes, the peak of *ν*(N−H) has been observed at 2842–2830 cm^−1^, which described no prominent change hence, deprotonation of ligands was not observed. Sharp and strong bands in continuous study of the spectra were observed as prominent peaks as *ν*(M−N) [31], *ν*(M−S) [32] at 560–520 and at 460–400 cm^−1^, respectively.

## 5. ELECTRONIC SPECTRA

All of the formed complexes have been found to be in +3 oxidation state. Ruthenium(III) complexes act as paramagnetic one and rhodium(III) complexes are diamagnetic. Electronic spectral data are given in Table 3. The ground state of ruthenium(III) is ^2^T_2*g*_ and the first excited doublet levels in order of increasing energy are ^2^A_2g_ and ^2^T_1g_ which are known to arise from t_2*g*_^4^e^1^_*g*_ configuration [33]. The ruthenium(III) complexes display electronic spectra with transition at 13500–14000 cm^−1^, 17240–18300 cm^−1^, and 23280–23800 cm^−1^ which may be assigned to ^2^T_2g_ → ^4^T_1g_, ^2^T_2g_ → ^4^T_2g_, and ^2^T_2g_ → ^2^A_2g_, ^2^T_1g_ in increasing order of energy. The B, C, and 10 Dq parameters were calculated using the following equations [34]:

(1) ^2^T_2g_ (t^5^) = 0, ^4^T_1g_ (t^4^ e) = 10 Dq − 5B − 4C, ^4^T_2g_ (t^4^ e) = 10 Dq + 3B − 4C, ^2^A_2g_, ^2^T_1g_ (t^4^ e) = 10 Dq − 2B − C.

The values of these ligand field parameters are comparable to those reported for other trivalent ruthenium complexes involving nitrogen, sulphur donor molecules [35]. The values are *ca.* 70–90% of the free ion values. The considerable decrease in the Racah interelectronic repulsion parameter, B, suggests the presence of strong covalent bonding between the donor and the metal ions. The overall effect will be an increase in the observed Dq value; high Dq values are usually associated with considerable electron delocalization [36]. Rhodium(III) complexes exhibit electronic spectra with transitions at 17260–17650 cm^−1^, 20210–20960 cm^−1^, and 27170–28590 cm^−1^. These bands resemble to those of reported transitions for other hexacoordinated rhodium complexes [37]. The ground state for rhodium(III) ion is ^1^A_1g_ in octahedral field, although in many instances only ^1^A_1g_ → ^1^T_1g_ spin allowed ligand field transitions to be observed. These transitions correspond to the ^1^A_1g_ → ^3^T_1g_, ^1^A_1g_ → ^1^T_1g_, and ^1^A_1g_ → ^1^T_2g_, respectively, which agree well with an octahedral geometry. The B and 10 Dq values were calculated from the positions of their electronic bands using the following equations:

(2) $$\begin{array}{c} \nu_{1}=10\;\text{D}\text{q}-4\text{B}+\frac{86{\left(\text{B}\right)}^{2}}{10\;\text{D}\text{q}},\\ \nu_{2}=10\;\text{D}\text{q}+12\text{B}+\frac{2{\left(\text{B}\right)}^{2}}{10\;\text{D}\text{q}}. \end{array}$$

The ratios of the energies of *ν*_2_ and *ν*_1_ are in the range 1.32–1.37. The B values are 57–67% of the free ion value. The decrease in B values from the free ion value suggests that there is a considerable orbital overlap with strong covalency in the metal ligand *σ* bond [38].

## 6. MAGNETIC MOMENTS

The room temperature magnetic moments of all the ruthenium(III) thiosemicarbazone complexes lie in the range 1.08–1.90 B.M., which are expected to be lower than the predicted value of 2.10 B.M. This lowering may occur due to the presence of lower symmetry ligand fields, metal-metal interactions, or extensive electron delocalization in species [39]. Rhodium(III) complexes are diamagnetic and, as expected, this is again consistent with octahedral geometry of nitrogen and sulphur atoms producing a strong field [40].

### 6.1. ^1^H and ^13^C NMR

Coordination of thiosemicarbazones in the rhodium(III) complexes are further confirmed by ^1^ H and ^13^ C NMR spectra (see Table 4). The resonance for methoxy protons appeared as a singlet at *δ* 3.65 ppm in ligands and in complexes no significant change was observed. Significant azomethine proton signal, due to CH=N, was observed at *δ* 8.02–9.02 ppm region as a multiplet in ligands, and in complexes it has shown a change as a downfield shift and occurred at *δ* 8.20–9.20 ppm, indicating involvement of nitrogen in coordination. The proton peak of N−H group at *δ* 10.6–11.2 ppm remains the same in the ligands, and in the complexes it suggested that deprotonation do not occur and it has also shown keto form of the ligands. The multiplets as strong bands in region *δ* 6.2–8.2 ppm were assigned to aromatic ring protons, which also shifted downfield in the complexes.

The ^13^ C NMR spectra revealed the presence of expected number of signals corresponding to different types of carbon atoms present in the compounds. In ligands as well as in complexes, −OCH_3_ group absorbs at *δ* 65.0–65.2 ppm and at *δ* 65.5–65.6 ppm slightly downfield to the methyl group carbon due to the deshielding of the directly attached electronegative oxygen atom. No change on complexation to this group occurs. The spectra of the ligands exhibit a strong band at *δ* 179.2–180.2 ppm and are assigned as C=S group. This band undergoes upfield shift of *δ* 7.2–7.4 ppm and occurs at *δ* 171.9–172.8 ppm. This has shown involvement of thione sulphur in coordination. The signals due to azomethine carbon occurred at *δ* 162.3–165.2 ppm as downfield peak, and on complexation they have shown shift to *δ* 160.5–163.0 ppm due to the resonance and also have given proof that nitrogen is involved in coordination.

## 7. THERMAL STUDIES

The TGA data reveal that there is a good agreement with the formulae as suggested from the elemental analyses. The first mass loss occurs within the temperature range 190–300°C, which corresponds to the removal of three chloride ions of the outer sphere as HCl. The number of chelate rings as well as the type of chelate rings around metal ions play an important role in the thermal stability and degradation of the complexes. Furthermore, it is known that the electronegativity and atomic radius of the central metal also affect the thermal stability. No endothermic peak has been observed, indicating absence of water molecule. Thermal investigations of [Ru(HAPT)_3_]Cl_3_ support the removal of the organic part of the ligand as PhNHCS fragments in the temperature range 320–360°C. The third step corresponds to the removal of the three molecules of C_7_H_5_OCH_3_ at temperature range 400–480°C. Final decomposition leaves a mixed residue of Ru_2_O_3_−RuO_2_ at 680–695°C. The same decomposition pattern was observed for other complexes of ruthenium and rhodium leaving residues of RuO_2_ and Rh_2_O_3_, respectively, in the temperature range 710–750°C like a carbonaceous matter.

## 8. ANTIBACTERIAL STUDIES

The results (Table 5) exhibit that complexes show moderate activity against *Bacillus subtilis* and *Pseudomonos aeruginosa*. The toxicity of the complexes was found better than parent ligand owing to the chelation theory of Tweedy [41]. The [Ru(HVPT)_3_]Cl_3_, [Ru(HAPT)_3_]Cl_3_, and [Ru(HVNPT)_3_]Cl_3_ exhibited higher toxicity; this is due to the presence of electron donating group (OCH_3_) in these complexes while in the same complexes of rhodium better toxicity was also observed. The variation in the toxicity of different complexes against various organisms depends either on the impermeability of the cells of the microbes or differences in ribosome in microbial cells [42]. The enhanced effect of complexes due to chelation could increase the lipophilicity of the central metal atom, which favours the permeation through the lipid layers of the cell wall. On the other hand, the mode of action of the compounds may involve the formation of hydrogen bonds through azomethine group of the complexes with the active centers of cell constituents resulting in the interference with normal cell process. Besides, antibacterial activity could not reach the affectivity of the streptomycin. On the basis of the above studies, the structures in Figure 2 may be formulated for the complexes.

## Figures and Tables

**Table 1:** Analytical data for the ligands and their Ru(III) and Rh(III) complexes.

Compounds	M. wt. found (calcd.)	Yield (%)	Color	Analysis: found (calcd.)%						*μ*_eff_ BM

				C	H	N	Cl/F	S	M	

HAPT	285	70	Pale yellow	63.0	5.0	14.2	—	11.0	—	—
	(285)			(63.1)	(5.2)	(14.7)		(11.2)		
HANPT	328	72	Yellow brown	54.3	3.9	16.2	—	9.5	—	—
	(330)			(54.5)	(4.2)	(16.9)		(9.6)		
HCBPT	288	70	Cream yellow	57.8	3.9	14.0	12.0	11.0	—	—
	(289)			(58.1)	(4.1)	(14.5)	(12.1)	(11.0)		
HCBNPT	332	75	Yellow	49.0	3.0	16.2	10.0	9.3	—	—
	(334)			(50.2)	(3.2)	(16.7)	(10.4)	(9.5)		
HFBPT	271	74	Yellow	61.0	4.0	15.0	6.2	11.4	—	—
	(273)			(61.4)	(4.3)	(15.3)	(6.9)	(11.7)		
HFBNPT	317	75	Yellow	52.6	3.0	17.2	5.4	10.0	—	—
	(318)			(52.8)	(3.4)	(17.6)	(5.9)	(10.0)		
HVPT	300	75	Yellow brown	59.1	4.7	13.8	—	10.4	—	—
	(301)			(59.7)	(4.9)	(13.9)		(10.6)		
HVNPT	345	75	Yellow	51.5	3.8	15.9	—	9.2	—	—
	(346)			(51.9)	(4.0)	(16.1)		(9.2)		
[Ru(HAPT)_3_]Cl_3_	1062	68	Greenish brown	50.2	3.9	11.6	****9.6	9.0	9.2	1.08
	(1063)			(50.7)	****(4.2)	(11.8)	(9.8)	(9.0)	(9.5)	
[Ru(HANPT)_3_]Cl_3_	1196	60	Black	44.9	3.2	13.8	8.5	8.0	8.2	1.88
	(1197)			(45.0)	(3.5)	(14.0)	(8.7)	(7.9)	(8.4)	
[Ru(HCBPT)_3_]Cl_3_	1075	60	Brown	46.4	3.0	11.2	19.3	8.9	9.0	1.89
	(1076)			(46.8)	(3.3)	(11.7)	(19.5)	(8.6)	(9.3)	
[Ru(HCBNPT)_3_]Cl_3_	1210	62	Black	41.3	2.5	13.2	17.1	7.9	8.0	1.78
	(1205)			(41.6)	(2.7)	(13.8)	(17.3)	(7.6)	(8.3)	
[Ru(HFBPT)_3_]Cl_3_	1026	60	Dark brown	48.0	2.6	12.0	10.0	9.3	9.2	1.80
	(1027)			(49.1)	(3.5)	(12.2)	(10.2)	(9.3)	(9.8)	
[Ru(HFBNPT)_3_]Cl_3_	1160	60	Black	43.1	2.6	14.2	8.8	8.2	8.4	1.90
	(1162)			(43.4)	(2.8)	(14.4)	(9.0)	(8.0)	(8.6)	
[Ru(HVPT)_3_]Cl_3_	1110	60	Brown	48.2	3.7	11.1	9.2	8.6	8.9	1.68
	(1111)			(48.6)	(4.0)	(11.3)	(9.4)	(8.5)	(9.0)	
[Ru(HVNPT)_3_]Cl_3_	1243	65	Black	43.0	3.1	13.2	8.2	7.7	7.9	1.70
	(1245)			(43.3)	(3.3)	(13.4)	(8.4)	(7.2)	(8.1)	
[Rh(HAPT)_3_]Cl_3_	1063	70	Orange brown	50.2	3.9	11.2	9.4	9.0	9.0	—
	(1065)			(50.7)	(4.2)	(11.8)	(9.8)	(8.9)	(9.5)	
[Rh(HANPT)_3_]Cl_3_	1196	65	Maroon	44.8	3.3	13.9	8.5	8.0	8.2	—
	(1198)			(45.0)	(3.5)	(14.0)	(8.7)	(8.0)	(8.5)	
[Rh(HCBPT)_3_]Cl_3_	1076	60	Brown	46.5	3.0	11.2	19.2	8.9	9.3	—
	(1077)			(46.7)	(3.3)	(11.6)	(19.4)	(8.4)	(9.5)	
[Rh(HCBNPT)_3_]Cl_3_	1211	65	Brown	41.2	2.4	13.3	17.1	7.9	8.2	—
	(1213)			(41.5)	(2.7)	(13.8)	(17.3)	(7.4)	(8.4)	
[Rh(HFBPT)_3_]Cl_3_	1028	62	Rusty brown	48.9	3.1	11.8	10.0	9.3	9.8	—
	(1028)			(49.0)	(3.5)	(12.2)	(10.2)	****(9.0)	(10.0)	
[Rh(HFBNPT)_3_]Cl_3_	1162	62	Brown	43.1	2.6	14.3	8.9	8.2	8.6	—
	(1163)			(43.3)	(2.8)	(14.4)	(9.0)	(8.1)	(8.7)	
[Rh(HVPT)_3_]Cl_3_	1112	60	Maroon	48.4	3.9	11.2	9.3	8.6	9.0	—
	(1113)			(48.5)	(4.0)	(11.3)	(9.4)	(8.3)	(9.1)	
[Rh(HVNPT)_3_]Cl_3_	1244	68	Blackish brown	43.1	3.2	13.2	8.2	7.7	8.0	—
	(1245)			(43.3)	(3.3)	(13.4)	(8.4)	(7.2)	(8.1)	

**Table 2:** Infrared spectral data (cm^−1^) of the ligands and its complexes. s = strong, m = medium, w = weak.

Compounds	Assignments					

	*ν*(N−H)	*ν*(N−N)	*ν*(C=N)	*ν*(C=S)	*ν*(M−N)	*ν*(M−S)

HAPT	2831 s	1030 m	1594 s	827 s	—	—
HANPT	2832 s	1022 m	1600 s	860 s	—	—
HCBPT	2830 s	1020 m	1594 s	862 s	—	—
HCBNPT	2835 s	1028 m	1595 s	872 s	—	—
HFBPT	2840 s	1030 m	1610 s	830 s	—	—
HFBNPT	2835 s	1020 m	1595 s	870 s	—	—
HVPT	2842 s	1026 m	1605 s	860 s	—	—
HVNPT	2840 s	1022 m	1610 s	830 s	—	—
[Ru(HAPT)_3_]Cl_3_	2831 s	1036 m	1580 s	820 s	520 m	440 s
[Ru(HANPT)_3_]Cl_3_	2831 s	1030 m	1590 s	850 s	560 m	460 s
[Ru(HCBPT)_3_]Cl_3_	2830 s	1030 m	1585 s	852 s	535 s	400 s
[Ru(HCBNPT)_3_]Cl_3_	2834 m	1035 m	1580 s	860 s	520 m	430 s
[Ru(HFBPT)_3_]Cl_3_	2841 m	1040 m	1600 s	820 s	525 w	410 m
[Ru(HFBNPT)_3_]Cl_3_	2835 s	1025 m	1585 m	860 s	530 m	450 m
[Ru(HVPT)_3_]Cl_3_	2842 s	1032 m	1590 m	850 s	545 m	430 m
[Ru(HVNPT)_3_]Cl_3_	2841 s	1033 m	1600 s	820 s	560 m	435 s
[Rh(HAPT)_3_]Cl_3_	2830 m	1035 m	1580 s	820 s	520 m	430 w
[Rh(HANPT)_3_]Cl_3_	2832 s	1032 m	1589 m	840 s	530 w	450 w
[Rh(HCBPT)_3_]Cl_3_	2831 m	1032 m	1580 s	850 s	540 w	445 w
[Rh(HCBNPT)_3_]Cl_3_	2836 s	1035 m	1585 s	855 s	560 w	418 m
[Rh(HFBPT)_3_]Cl_3_	2841 s	1038 m	1600 s	820s	545 m	415 m
[Rh(HFBNPT)_3_]Cl_3_	2835 w	1028 m	1580 s	860 s	542 m	425 s
[Rh(HVPT)_3_]Cl_3_	2842 s	1036 m	1592 m	845 s	535 m	435 s
[Rh(HVNPT)_3_]Cl_3_	2841 s	1030 m	1598 s	820 s	532 m	440 m

**Table 3:** Electronic spectral bands (cm^−1^) and ligand field parameters of the Ru(III) and Rh(III) complexes.

Complex	*λ*_max_ (cm^−1^)	Assignments	*ν*_2_/*ν*_1_	10 Dq (cm^−1^)	B (cm^−1^)	C (cm^−1^)	*β*

[Ru(HAPT)_3_]Cl_3_	13700	^2^ T_2g_ → ^4^ T_1g_ (*ν*_1_)	1.25	27342	443	2858	0.70
	17240	^2^ T_2g_ → ^4^ T_2g_ (*ν*_2_)					
	23600	^2^ T_2g_ → ^2^ A_2g_, ^2^ T_1g_ (*ν*_3_)					

[Ru(HANPT)_3_]Cl_3_	13500	-do-	1.27	27383	470	2883	0.75
	17260						
	23560						

[Ru(HCBPT)_3_]Cl_3_	14060	-do-	1.27	27255	475	2705	0.76
	17860						
	23600						

[Ru(HCBNPT)_3_]Cl_3_	13520	-do-	1.28	27352	474	2866	0.75
	17310						
	23540						

[Ru(HFBPT)_3_]Cl_3_	13620	-do-	1.31	27277	539	2741	0.86
	17930						
	23460						

[Ru(HFBNPT)_3_]Cl_3_	14000	-do-	1.30	27309	538	2656	0.86
	18300						
	23580						

[Ru(HVPT)_3_]Cl_3_	13510	-do-	1.33	27795	565	2865	0.90
	18030						
	23800						

[Ru(HVNPT)_3_]Cl_3_	14060	-do-	1.29	26875	523	2551	0.83
	18240						
	23280						

[Rh(HAPT)_3_]Cl_3_	17600	^1^ A_1g_ → ^3^ T_1g_	1.34	21950	435	1740	0.60
	20210	^1^ A_1g_ → ^1^ T_1g_ (*ν*_1_)					
	27170	^1^ A_1g_ → ^1^ T_2g_ (*ν*_2_)					

[Rh(HANPT)_3_]Cl_3_	17550	-do-	1.35	22060	445	1780	0.62
	20280						
	27400						

[Rh(HCBPT)_3_]Cl_3_	17260	-do-	1.32	22615	413	1655	0.57
	20960						
	27580						

[Rh(HCBNPT)_3_]Cl_3_	17300	-do-	1.35	21990	442	1770	0.61
	20220						
	27300						

[Rh(HFBPT)_3_]Cl_3_	17400	-do-	1.37	22290	478	1910	0.66
	20380						
	28020						

[Rh(HFBNPT)_3_]Cl_3_	17640	-do-	1.37	22815	481	1925	0.67
	20890						
	28590						

[Rh(HVPT)_3_]Cl_3_	17650	-do-	1.32	22618	414	1658	0.58
	20960						
	27590						

[Rh(HVNPT)_3_]Cl_3_	17460	-do-	1.34	22655	444	1775	0.62
	20880						
	27980						

**Table 4:** NMR spectral data (*δ*, ppm) of the thiosemicarbazones and their rhodium(III) complexes.

Compounds	^1^H−				^13^C−		

	*δ*(CH=N)	*δ*(N−H)	*δ*(Ar−H)	*δ*(OCH_3_)	*δ*(C=N)	*δ*(C=S)	*δ*(O−CH_3_)

HAPT	8.02 (s)	10.9 (s)	6.2–7.0 (m)	3.62 (s)	162.3	179.2	65.0
HANPT	8.06 (s)	11.0 (s)	6.2–7.8 (m)	3.65 (s)	162.6	179.6	65.2
HCBPT	8.08 (s)	11.0 (s)	6.2–7.6 (m)	—	163.2	180.0	—
HCBNPT	8.06 (s)	11.2 (s)	6.2–7.8 (m)	—	163.8	180.3	—
HFBPT	9.00 (s)	10.9 (s)	6.3–7.2 (m)	—	165.0	179.8	—
HFBNPT	9.02 (s)	11.1 (s)	6.6–8.0 (m)	—	165.2	180.2	—
HVPT	9.02 (s)	11.0 (s)	6.8–8.0 (m)	3.60 (s)	164.2	179.5	65.0
HVNPT	8.08 (s)	11.2 (s)	6.2–7.8 (m)	3.61 (s)	163.9	180.2	65.2
[Rh(HAPT)_3_]Cl_3_	8.20 (s)	10.6 (s)	6.4–7.2 (m)	3.65 (s)	160.5	171.9	65.5
[Rh(HANPT)_3_]Cl_3_	8.28 (s)	10.9 (s)	6.5–7.6 (m)	3.66 (s)	160.8	172.2	65.5
[Rh(HCBPT)_3_]Cl_3_	8.26 (s)	11.1 (s)	6.6–7.9 (m)	—	162.0	172.6	—
[Rh(HCBNPT)_3_]Cl_3_	9.20 (s)	11.0 (s)	6.8–8.2 (m)	—	162.6	172.8	—
[Rh(HFBPT)_3_]Cl_3_	9.18 (s)	10.9 (s)	6.9–8.0 (m)	—	163.0	172.6	—
[Rh(HFBNPT)_3_]Cl_3_	9.20 (s)	11.0 (s)	6.6–8.0 (m)	—	163.0	172.8	—
[Rh(HVPT)_3_]Cl_3_	9.16 (s)	11.2 (s)	6.9–7.2 (m)	3.62 (s)	161.6	172.3	65.6
[Rh(HVNPT)_3_]Cl_3_	8.20 (s)	11.0 (s)	6.4–7.8 (m)	3.61 (s)	161.2	172.8	65.6

**Table 5:** Antibacterial screening data of thiosemicarbazones and their Ru(III) and Rh(III) complexes.

Compounds	Inhibition zone (*μ*gmL^−1^)			

	Bacillus subtilis		Pseudomonos aeruginosa	

	500	1000	500	1000

HAPT	7	9	8	9
[Ru(HAPT)_3_]Cl_3_	14	17	13	16
[Rh(HAPT)_3_]Cl_3_	10	12	9	12
HANPT	7	10	7	11
[Ru(HANPT)_3_]Cl_3_	12	16	11	16
[Rh(HANPT)_3_]Cl_3_	10	14	10	13
HCBPT	6	10	6	9
[Ru(HCBPT)_3_]Cl_3_	13	16	12	16
[Rh(HCBPT)_3_]Cl_3_	11	16	11	14
HCBNPT	7	10	7	11
[Ru(HCBNPT)_3_]Cl_3_	14	18	15	19
[Rh(HCBNPT)_3_]Cl_3_	12	14	13	15
HFBPT	7	9	6	10
[Ru(HFBPT)_3_]Cl_3_	13	16	13	17
[Rh(HFBPT)_3_]Cl_3_	10	13	10	12
HFBNPT	6	10	6	9
[Ru(HFBNPT)_3_]Cl_3_	14	17	13	16
[Rh(HFBNPT)_3_]Cl_3_	11	14	11	15
HVPT	9	12	9	11
[Ru(HVPT)_3_]Cl_3_	14	17	14	16
[Rh(HVPT)_3_]Cl_3_	11	14	11	13
HVNPT	8	10	9	11
[Ru(HVNPT)_3_]Cl_3_	15	18	14	18
[Rh(HVNPT)_3_]Cl_3_	12	15	12	14
Streptomycin	17	18	21	22

## References

[1] Mishra D, Naskar S, Drew MGB, Chattopadhyay SK (2006). Synthesis, spectroscopic and redox properties of some ruthenium(II) thiosemicarbazone complexes: structural description of four of these complexes. *Inorganica Chimica Acta*.

[2] Casas JS, García-Tasende MS, Sordo J (2000). Main group metal complexes of semicarbazones and thiosemicarbazones. A structural review. *Coordination Chemistry Reviews*.

[3] Padhyé S, Kauffman GB (1985). Transition metal complexes of semicarbazones and thiosemicarbazones. *Coordination Chemistry Reviews*.

[4] Pal I, Basuli F, Bhattacharya S (2002). Thiosemicarbazone complexes of the platinum metals. A story of variable coordination modes. *Proceedings of the Indian Academy of Sciences: Chemical Sciences*.

[5] Belicchi Ferrari M, Capacchi S, Pelosi G (1999). Synthesis, structural characterization and biological activity of helicin thiosemicarbazone monohydrate and a copper(II) complex of salicylaldehyde thiosemicarbazone. *Inorganica Chimica Acta*.

[6] Dutta S, Basuli F, Peng S-M, Lee G-H, Bhattacharya S (2002). Synthesis, structure and redox properties of some thiosemicarbazone complexes of rhodium. *New Journal of Chemistry*.

[7] El-Sawaf AK, West DX, El-Saied FA, El-Bahnasawy RM (1998). Synthesis, magnetic and spectral studies of iron(III), cobalt(II, III), nickel(II), copper(II) and zinc(II) complexes of 4-formylantipyrine N(4)-antipyrinylthiosemicarbazone. *Transition Metal Chemistry*.

[8] Purohit S, Koley AP, Prasad LS, Manoharan PT, Ghosh S (1989). Chemistry of molybdenum with hard-soft donor ligands. 2. Molybdenum(VI), -(V), and -(IV) oxo complexes with tridentate Schiff base ligands. *Inorganic Chemistry*.

[9] West DX, Swearingen JK, Valdés-Martínez J (1999). Spectral and structural studies of iron(III), cobalt(II,III) and nickel(II) complexes of 2-pyridineformamide N(4)-methylthiosemicarbazone. *Polyhedron*.

[10] Afrasiabi Z, Sinn E, Chen J (2004). Appended 1,2-naphthoquinones as anticancer agents 1: synthesis, structural, spectral and antitumor activities of ortho-naphthaquinone thiosemicarbazone and its transition metal complexes. *Inorganica Chimica Acta*.

[11] Kovala-Demertzi D, Miller JR, Kourkoumelis N, Hadjikakou SK, Demertzis MA (1999). Palladium(II) and platinum(II) complexes of pyridine-2-carbaldehyde thiosemicarbazone with potential biological activity. Synthesis, structure and spectral properties. Extended network via hydrogen bond linkages of [Pd(PyTsc)Cl]. *Polyhedron*.

[12] Singh NK, Singh SB (2001). Synthesis, characterization and biological properties of manganese(II), cobalt(II), nickel(II), copper(II), zinc(II), chromium(III) and iron(III) complexes with a new thiosemicarbazide derivative. *Indian Journal of Chemistry*.

[13] Agarwal RK, Singh L, Sharma DK (2006). Synthesis, spectral, and biological properties of copper(II) complexes of thiosemicarbazones of Schiff bases derived from 4-aminoantipyrine and aromatic aldehydes. *Bioinorganic Chemistry and Applications*.

[14] García-Tojal J, Lezama L, Pizarro JL, Insausti M, Arriortua MI, Rojo T (1999). Spectroscopic and magnetic properties of copper(II) complexes derived from pyridine-2-carbaldehyde thiosemicarbazone. Structures of 
[Cu(NO_3_)(C_7_ H_8_N_4_S)(H_2_O)](NO_3_) and [{Cu(NCS)(C_7_H_7_N_4_S)}_2_]. *Polyhedron*.

[15] Labisbal E, Haslow KD, Sousa-Pedrares A, Valdés-Martínez J, Hernández-Ortega S, West DX (2003). Copper(II) and nickel(II) complexes of 5-methyl-2-hydroxyacetophenone *N*(4)-substituted thiosemicarbazones. *Polyhedron*.

[16] El-Shazly RM, Al-Hazmi GAA, Ghazy SE, El-Shahawi MS, El-Asmy AA (2005). Spectroscopic, thermal and electrochemical studies on some nickel(II) thiosemicarbazone complexes. *Spectrochimica Acta—Part A: Molecular and Biomolecular Spectroscopy*.

[17] Mostafa SI, El-Asmy AA, El-Shahawi MS (2000). Ruthenium(II) 2-hydroxybenzophenone N(4)-substituted thiosemicarbazone complexes. *Transition Metal Chemistry*.

[18] Chattopadhyay SK, Ghosh SA (1987). A study of Ru(II) complexes of some selected N—S donors. *Inorganica Chimica Acta*.

[19] Mukkanti K, Singh RP (1987). Complexes of platinum, rhodium, iridium and ruthenium with a thiosemicarbazone derived from thiophene-2-carboxaldehyde. *Transition Metal Chemistry*.

[20] Sinha PK, Chakravarty J, Bhattacharya S (1997). Synthesis, characterization, redox properties and reactivities of a group of phenolato complexes of ruthenium(III). *Polyhedron*.

[21] Chakravarty J, Bhattacharya S (1994). Synthesis, characterization, electron-transfer properties and reactivities of a group of ruthenium(II) complexes with 
RuN_2_P_2_X_2_(X = Cl, Br) coordination spheres. *Polyhedron*.

[22] Chattopadhyay SK, Hossain M, Ghosh S, Guha AK (1990). Ligational behaviour of two biologically active N—S donors towards cobalt(III), iron(III), iron(II) and rhodium(III). *Transition Metal Chemistry*.

[23] Jayabalakrishnan C, Karvembu R, Natarajan K (2002). Synthesis, characterisation, catalytic, and biocidal studies of ruthenium(III) complexes with thiosemicarbazones of *β*-diketoesters. *Synthesis and Reactivity in Inorganic, Metal-Organic, and Nano-Metal Chemistry*.

[24] Collins CH, Lyne PM (1970). *Microbial Methods*.

[25] Sengupta P, Dinda R, Ghosh S (2002). Ruthenium(II) complexes of NSO donor ligands in the form of ring-substituted 4-phenyl-thiosemicarbazones of salicylaldehyde and o-hydroxyacetophenone. *Transition Metal Chemistry*.

[26] Vogel AI (1986). *A Textbook of Quantitative Inorganic Analyses*.

[27] Deepa KP, Aravindakshan KK (2000). Synthesis, characterization and thermal studies of thiosemicarbazones of N-methyl- and N-ethylacetoacetanilide. *Synthesis and Reactivity in Inorganic, Metal-Organic, and Nano-Metal Chemistry*.

[28] Joseph M, Sreekanth A, Suni V, Kurup MRP (2006). Spectral characterization of iron(III) complexes of 2-benzoylpyridine N(4)-substituted thiosemicarbazones. *Spectrochimica Acta—Part A: Molecular and Biomolecular Spectroscopy*.

[29] Offiong OE, Martelli S (1997). Stereochemistry and antitumour activity of platinum metal complexes of 2-acetylpyridine thiosemicarbazones. *Transition Metal Chemistry*.

[30] Chandra S, Gupta LK (2005). EPR, mass, IR, electronic, and magnetic studies on copper(II) complexes of semicarbazones and thiosemicarbazones. *Spectrochimica Acta—Part A: Molecular and Biomolecular Spectroscopy*.

[31] Sharma VK, Srivastava S, Srivastava A (2006). Novel coordination complexes of the trivalent ruthenium, rhodium and iridium with hydrazones derived from Isatin hydrazide and various aldehydes with spectral and biological characterization. *Polish Journal of Chemistry*.

[32] El-Tabl AS, Ayad MI (2003). Investigation of thiosemicarbazones as chelating agents. Synthesis and spectroscopic characterization of some new ruthenium(III) complexes. *Synthesis and Reactivity in Inorganic, Metal-Organic, and Nano-Metal Chemistry*.

[33] Venkatachalam G, Maheswaran S, Ramesh R (2005). Synthesis, spectra, redox property and catalytic activity of ruthenium(III) Schiff base complexes. *Indian Journal of Chemistry*.

[34] Lever ABP (1984). Electronic spectra of d^n^ ions. *Inorganic Electronic Spectroscopy*.

[35] Chandra S (1992). Pd(II), Pt(II), Rh(III), Ir(III) and Ru(III) complexes of n-pentyl and n-hexyl ketone thiosemicarbazones. *Synthesis and Reactivity in Inorganic, Metal-Organic, and Nano-Metal Chemistry*.

[36] Jorgensen CK (1964). *Absorption Spectra and Chemical Bonding in Complexes*.

[37] Chandra S, Singh R (1988). Pd(II), Pt(II), Rh(III), Ir(III) and Ru(III) complexes of some nitrogen -oxygen donor ligands. *Indian Journal of Chemistry*.

[38] El-Dissouky A, Kasem A, El-Sonbati AZ (1987). Synthesis and characterization of some octahedral transition metal complexes with phenyl-2-picolylketone thiosemicarbazone. *Transition Metal Chemistry*.

[39] El-Saied FA, El-Bahnasawy RM, Abdel-Azeem M, El-Sawaf AK (1994). Synthesis, characterization and electrochemical properties of *β*-diketone complexes of ruthenium(III). *Polyhedron*.

[40] Sharma VK, Sengupta SK (1993). Synthesis and spectroscopic studies on ruthenium (III) and rhodium (III) derivatives with thiohydantoins. *Synthesis and Reactivity in Inorganic, Metal-Organic, and Nano-Metal Chemistry*.

[41] Thangadurai TD, Natarajan K (2002). Synthesis and characterisation of ruthenium(III) complexes containing dibasic tetradentate Schiff bases. *Indian Journal of Chemistry*.

[42] Sengupta SK, Pandey OP, Srivastava BK, Sharma VK (1998). Synthesis, structural and biochemical aspects of titanocene and zirconocene chelates of acetylferrocenyl thiosemicarbazones. *Transition Metal Chemistry*.

